# Supplementary material for: A novel autophagy enhancer as a therapeutic agent against metabolic syndrome and diabetes
Source: Nat Commun. 2018 Apr 12;9:1438. doi: 10.1038/s41467-018-03939-w (PMC5897400; doi:10.1038/s41467-018-03939-w)
Supplement: Supplementary file 1 — Supplementary Information [file 41467_2018_3939_MOESM1_ESM.pdf]

SUPPLEMENTARY INFORMATION

A novel autophagy enhancer as a therapeutic agent against metabolic syndrome and diabetes

Lim *et al.*

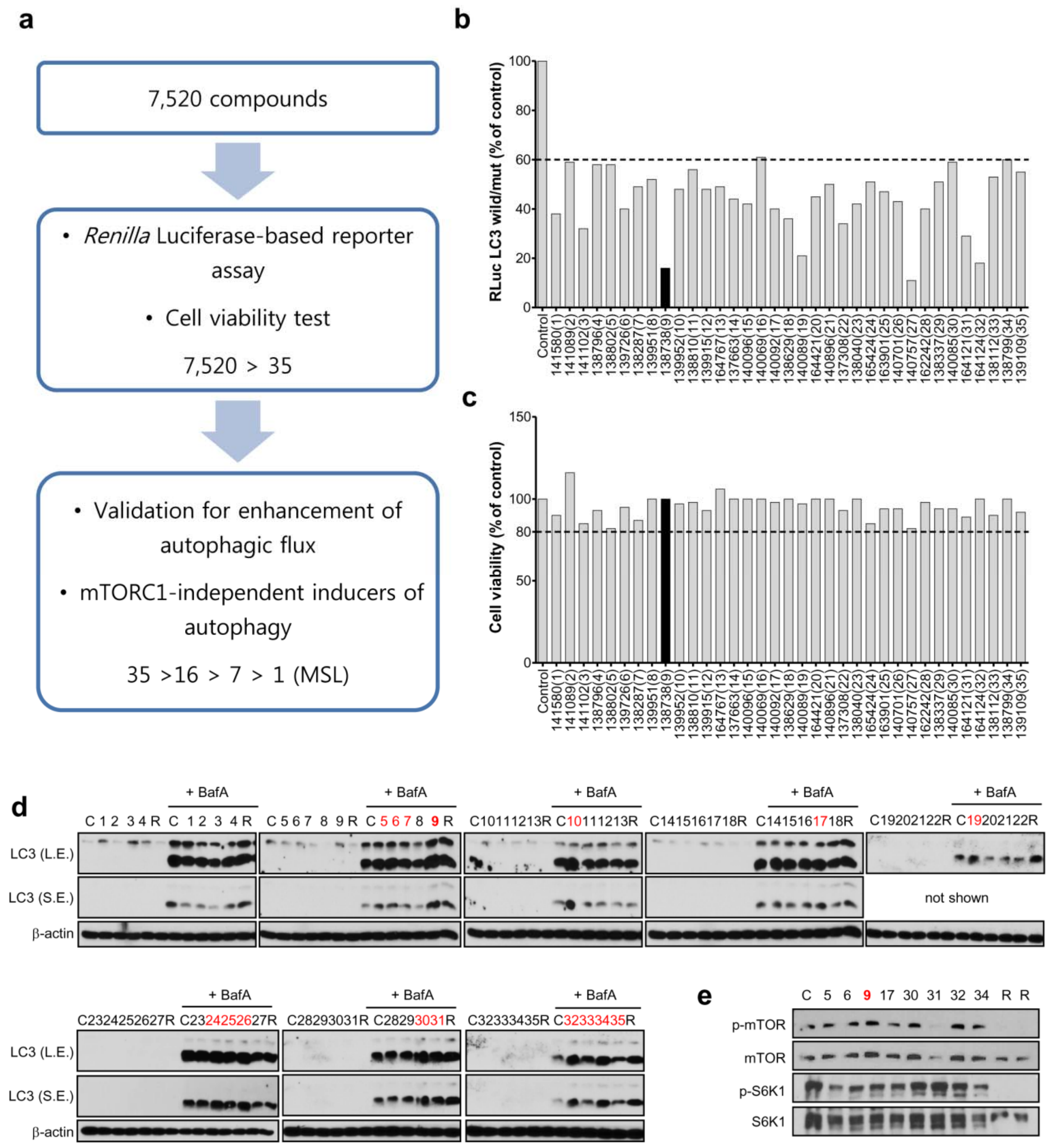

Supplementary Figure 1 Screening and selection of autophagy enhancer. (**a**) Scheme of autophagy enhancer screening. (**b**) HepG2 cells were stably transfected with wild-type p*RLuc*(C124A)-*LC3*(WT) or mutant p*RLuc*(C124A)-*LC3*(G120A) plasmid, and then treated with compounds from a chemical library. Chemicals that reduced normalized wild/mutant luciferase ratio to < 0.6 at 50 μM concentration were selected initially. The numbers in the parentheses are the serial numbers arbitrarily allocated to the chemicals that reduced

normalized wild/mutant luciferase ratio. The black bar indicates #9 ('MSL') that was used for experiments throughout the manuscript. (**c**) The 3-(4,5-dimethylthiazol-2-yl)-2,5-diphenyltetrazolium bromide (MTT) assay was conducted after treatment of transfectants with chemicals to exclude those with cytotoxic activity (< 80% viability). (**d**) SK-Hep1 cells were treated with selected chemicals of (**b**) and (**c**) at 50 μM concentration for 24 h with or without bafilomycin A1 (BafA). Treated cells were subjected to Western blot analysis using antibodies specific for LC3 or β-actin. The red numbers indicate chemicals that increased LC3-I to -II conversion in the presence of bafilomycin A1. (**e**) SK-Hep1 cells were treated with selected chemicals at 50 μM concentration for 24 h, and cell lysate was subjected to Western blot analysis using antibodies against phospho-mTOR, mTOR, phospho-S6K1 or S6K1. (C, control; R, rapamycin)

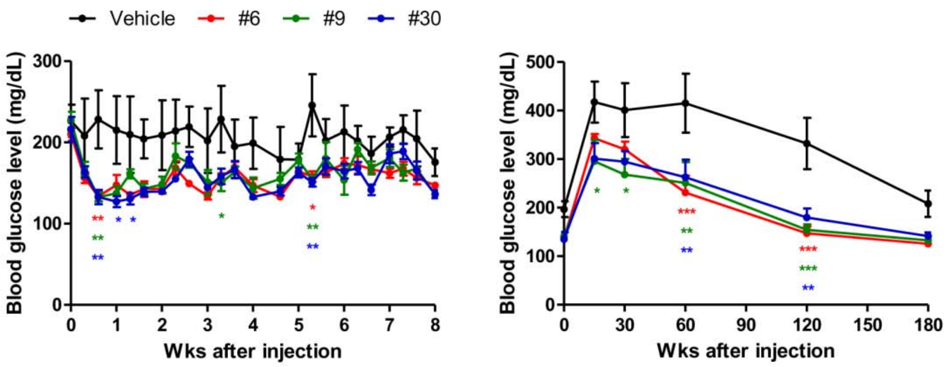

Supplementary Figure 2 Metabolic improvement by selected autophagy enhancer small molecules. Among the chemicals in **Supplementary Figure 1e**, 3 chemicals (#6, #9 and # 30) were administered to *ob/ob* mice for 8 weeks. Nonfasting blood glucose levels were monitored during *in vivo* administration of these chemicals (left) (F = 45.95, df = 3). Intraperitoneal glucose tolerance test (IPGTT) was conducted after *in vivo* administration of these chemicals for 8 weeks (right) (F = 4.965, df = 3). *P < 0.05, **P < 0.01 and ***P < 0.001 by two-way ANOVA with Bonferroni's post-hoc test.

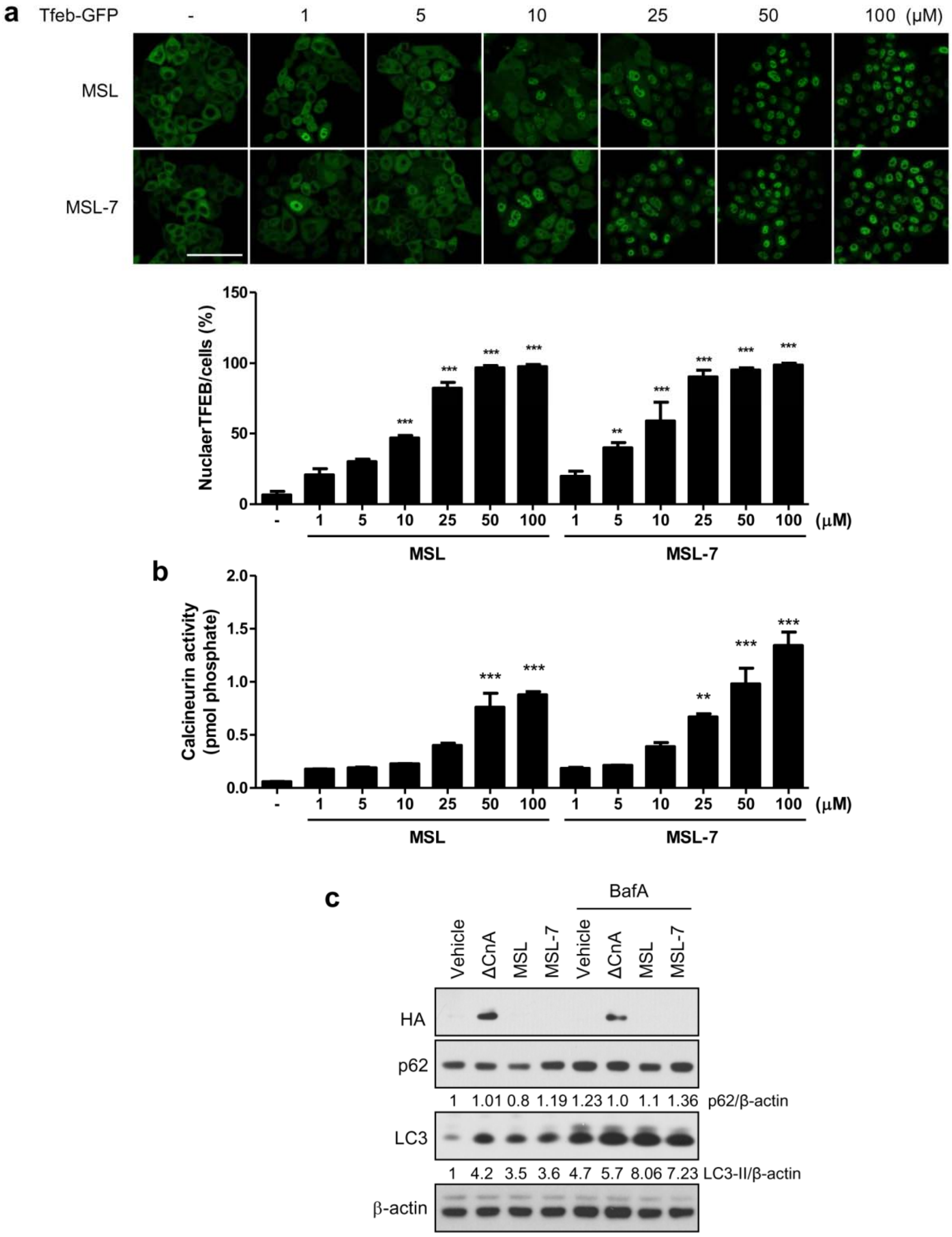

Supplementary Figure 3 Dose-response relationship between TFEB nuclear translocation or calcineurin activity and concentrations of MSL or MSL-7. (**a**) *Tfeb-GFP*-transfectant HeLa cells were treated with varying concentrations of MSL or MSL-7 for 2 h, and nuclear translocation of TFEB was determined by confocal microscopy (upper panel). The number of cells with nuclear TFEB was counted (lower panel) (F = 54.5, df treatment = 12, df residual =

24). (**b**) HeLa cells were treated with varying concentrations of MSL or MSL-7, and calcineurin activity was determined using a kit ($F = 33.9$, df treatment = 12, df residual = 13). (**c**) HeLa cells were transfected with *HA-ΔCaN* and *CnB* or treated with MSL or MSL-7 for 2 h. LC-3 conversion was determined by Western blot analysis using anti-p62 or -LC3 antibody in the presence or absence of bafilomycin A1 (BafA). Numbers below p62 or LC3-II immunoblot bands indicate fold changes normalized to β-actin bands. *$P < 0.05$, **$P < 0.01$ and ***$P < 0.001$ by one-way ANOVA with Tukey's post-hoc test (**a**,**b**).

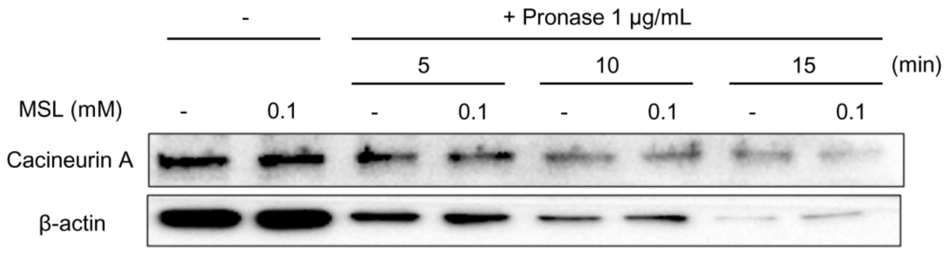

Supplementary Figure 4 Binding of MSL to calcineurin A. DARTS assay was conducted as in **Figure 2e** using a nonsaturating concentration of MSL (100 μM).

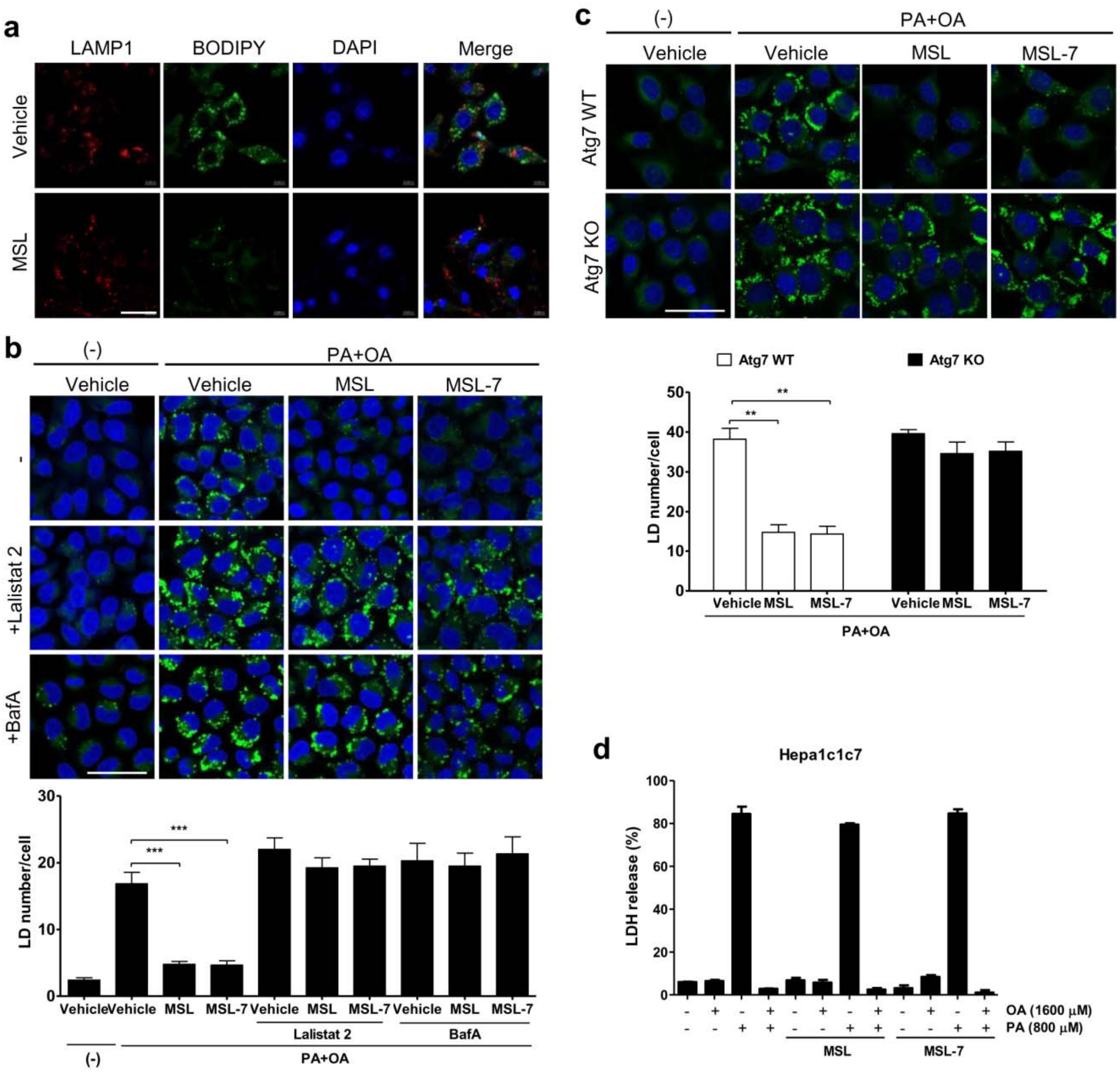

Supplementary Figure 5 Decreased intracellular lipid after treatment with autophagy enhancer. (**a**) HeLa cells loaded with PA (400 μM) + OA (800 μM) combination were treated with MSL for 24 h. After immunostaining with anti-LAMP1 antibody and BODIPY493/503 staining, confocal microscopy was conducted. (**b**) Cells loaded with PA + OA combination were treated with MSL in the presence or absence of lalistat 2 or bafilomycin A1 (BafA). After staining with BODIPY493/503, cells were subjected to confocal microscopy to determine the number of lipid droplets (LDs) (t = 6.53 for lalistat 2, t = 6.4 for bafilomycin A1, df = 17). (**c**) *Atg7* wild-type (WT) or knockout (KO) MEF cells loaded with PA + OA

combination were treated with MSL for 24 h. After BODIPY493/503 staining, confocal microscopy was conducted to count lipid droplet (LD) number ($t = 6.97$, $t = 7.08$, df = 8). (d) Hepa1c1c cells were treated with 800 μM PA with or without 1,600 μM OA for 24 h. LDH release was determined as a measure of lipotoxicity. **$P < 0.01$ and ***$P < 0.001$ two-tailed Student's *t*-test (**b**,**c**).

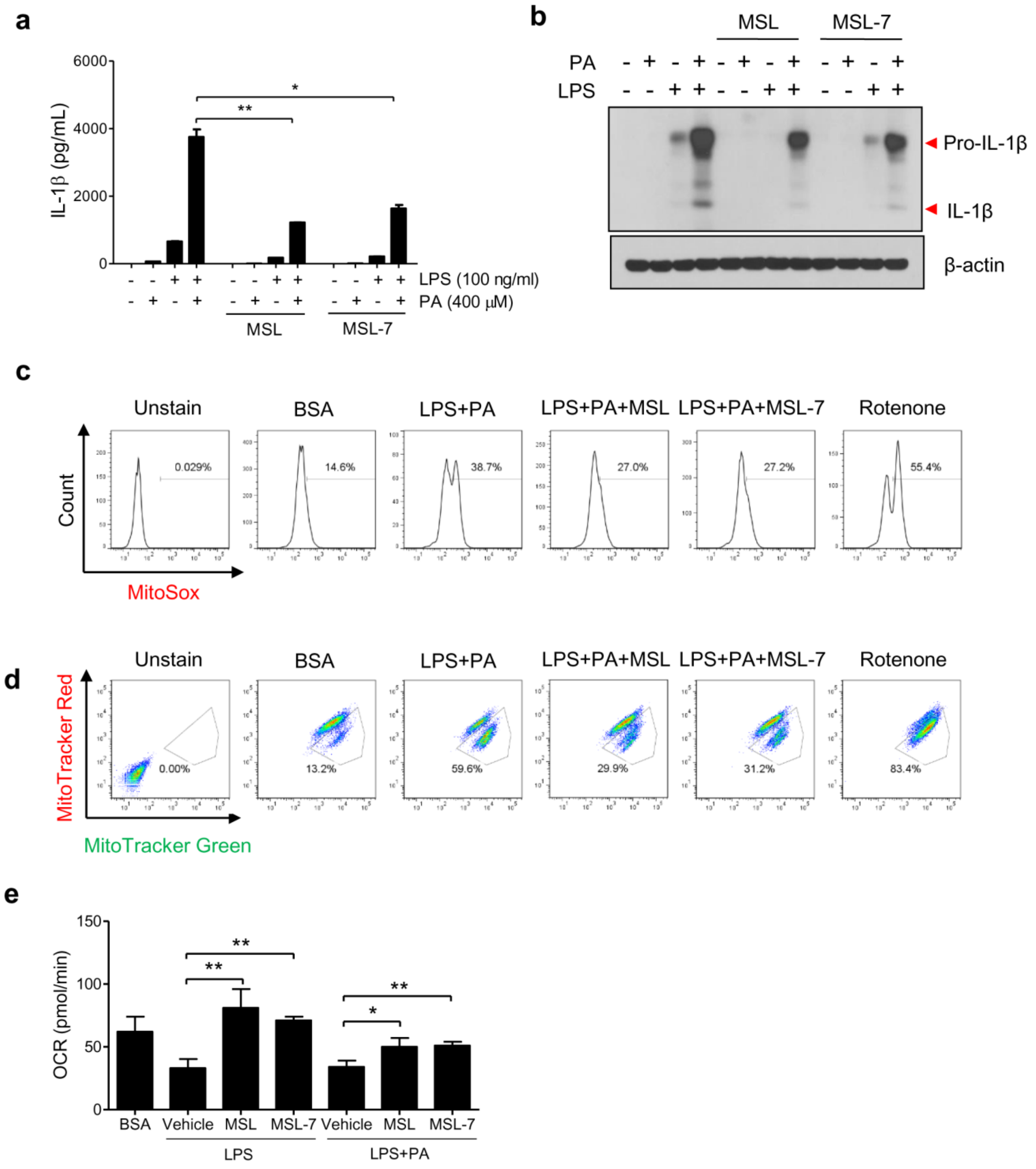

Supplementary Figure 6 Effect of autophagy enhancer on inflammasome activation and mitochondrial dysfunction. (**a**) Primary peritoneal macrophages were pretreated with MSL or MSL-7 for 24 h, and then incubated with 400 μM palmitic acid (PA) and/or 100 ng/ml LPS for additional 24 h. IL-1β in the culture supernatant was measured by ELISA (t = 35.35 for MSL, t = 8.587 for MSL-7, df = 2). (**b**) Primary peritoneal macrophages treated as in (**a**) were subjected to Western blot analysis using antibodies against IL-1β or β-actin. (**c**) After the

same treatment of macrophages as in (**a**), cells were incubated with MitoSox Red for FACS analysis of mitochondrial ROS as described in the Methods. Rotenone, a mitochondrial stressor, was employed as a positive control. (**d**) After the same treatment of macrophages as in (**a**), cells were incubated with MitoTracker Red and MitoTracker Green for FACS analysis of mitochondrial potential as described in the Methods. The numbers in (**c**) and (**d**) indicate the percentage of cells in the designated gate, i.e., cells with mitochondrial ROS and those with low mitochondrial potential, respectively. (**e**) ATP synthesis-coupled mitochondrial oxygen consumption rate (OCR) was determined using Seahorse XF Analyzer (F =10.98, df treatment = 6, df residual = 14). *$P < 0.05$, **$P < 0.01$ and ***$P < 0.001$ by one-way ANOVA with Tukey's post-hoc test (**e**) and two-tailed Student's *t*-test (**a**).

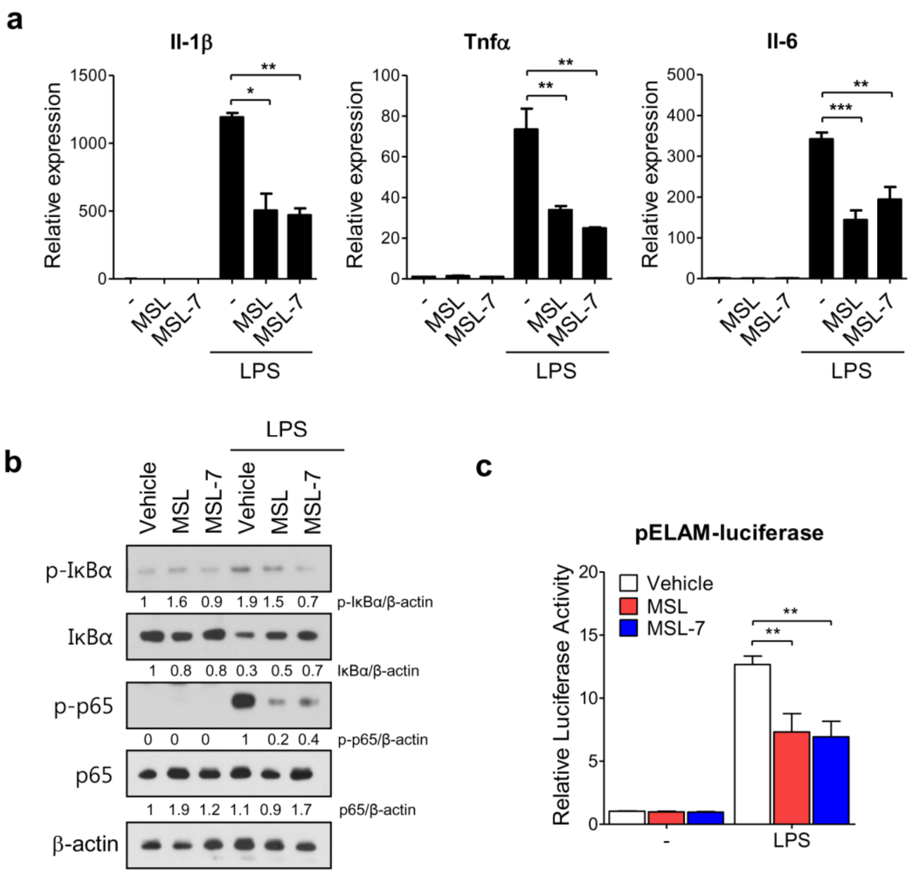

Supplementary Figure 7 NF-κB signaling in MSL-treated primary peritoneal macrophages. (**a**) After treatment of macrophages with LPS with or without pretreatment with MSL or MSL-7, mRNA was extracted and real-time RT-PCR was conducted using specific primer sets [t = 5.39 (MSL), t = 12.07 (MSL-7), df = 4 for *Il-1β*; t = 5.71 (MSL), t = 7.7 (MSL-7), df = 4 for *Tnfα*; t = 6.84 (MSL), t = 4.23 (MSL-7), df = 4 for *Il-6*]. (**b**) After the same treatment as in (**a**), cell extract was subjected to Western blot analysis using the indicated antibodies. (**c**) After transfection of macrophages with pELAM-luciferase plasmid, cells were treated with LPS with or without pretreatment with MSL or MSL-7. NF-κB reporter activity was measured using a microplate luminometer (Centro LB 960) [t = 4.59 (MSL), t = 3.55 (MSL-7), df = 8]. *$P < 0.05$, **$P < 0.01$ and ***$P < 0.001$ by two-tailed Student's t-test (**a**,**c**).

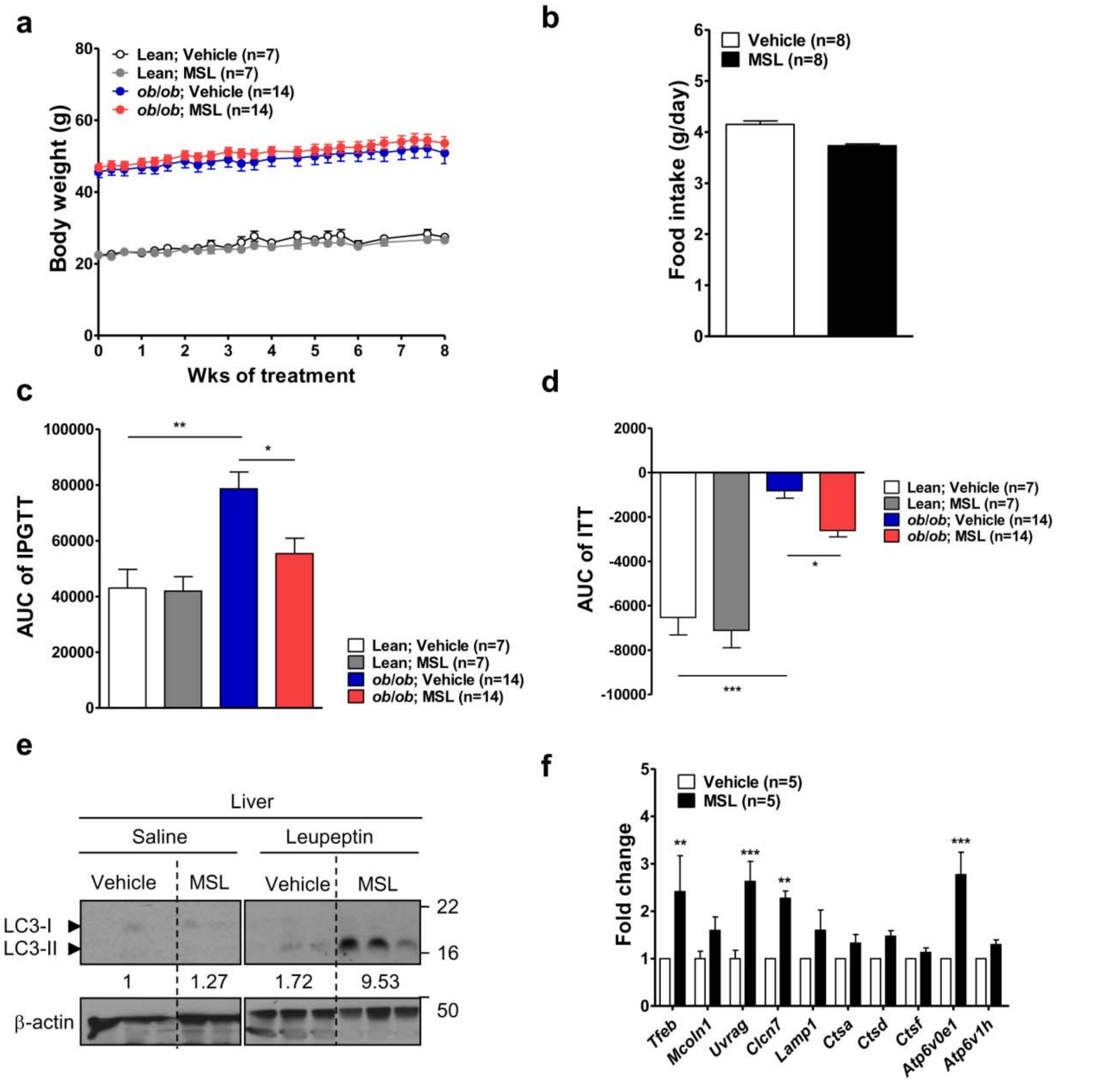

Supplementary Figure 8 *In vivo* administration of autophagy enhancer (MSL) to *ob/ob* mice. (**a**, **b**) Eight-week-old male *ob/ob* mice were treated with MSL (see **Figure 4**). During the observation period, body weight (**a**) and food intake (**b**) were monitored. (**c**) AUC of the IPGTT curve in **Figure 4c**. (F = 7.8, df treatment = 3, df residual = 38). (**d**) AUC of the ITT curve in **Figure 4d**. (F = 39.0, df treatment = 3, df residual = 38). (**e**) Liver tissue was obtained from *ob/ob* mice after MSL administration for 8 weeks, with or without 30 mg/kg leupeptin injection 1 h before sacrifice. Tissue lysate was subjected to Western blot analysis using antibodies against LC3 or β-actin. Numbers below LC3-II immunoblot bands indicate

fold changes normalized to β-actin bands. (**f**) mRNA was prepared from the liver tissue of (**e**), and real-time RT-PCR was conducted. (t = 4.1, df=8 for *Tfeb*; t = 4.7, df = 8 for *Uvrag*; t = 3.7, df = 8 for *Clcn7*; t = 5.1, df = 8 for *Atpv0E1*). All data in this figure are the means ± s.e.m. from ≥ 3 independent experiments performed in triplicate. *P < 0.05, **P < 0.01 and ***P < 0.001 by one-way ANOVA with Tukey's post-hoc test (**c**,**d**) or two-tailed Student's t-test (**f**).

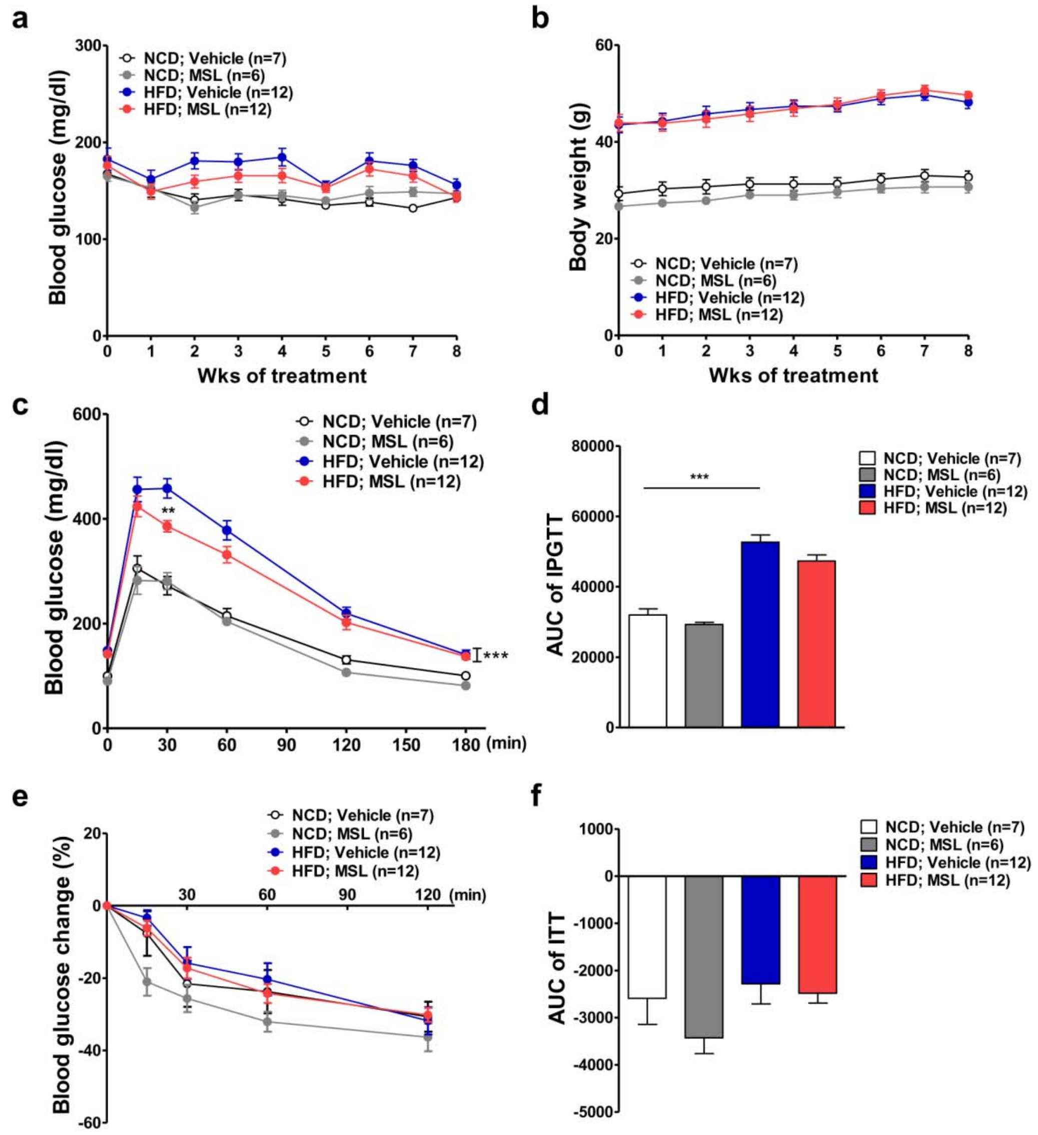

Supplementary Figure 9 *In vivo* administration of autophagy enhancer (MSL) to HFD-fed mice. Eight-week-old male C57BL/6 mice were fed HFD or normal chow diet (NCD) for 8 weeks, and then treated with MSL (50 mg/kg) 3 times a week for 8 weeks. Nonfasting blood glucose level (**a**) and body weight (**b**) were monitored. (**c**) IPGTT was conducted after *in vivo* administration of MSL for 8 weeks (F = 13.9, df = 1). (**d**) AUC of the IPGTT curve in (**c**). (F = 33.3, df treatment = 3, df residual = 33). (**e**) ITT was conducted after *in vivo* administration of MSL for 8 weeks. (**f**) AUC of the ITT curve in (**e**). All data in this figure are the means ± s.e.m. from ≥ 3 independent experiments performed in triplicate. $**P < 0.01$ and $***P <$

0.001 by two-way ANOVA with Bonferroni's post-hoc test (**c**) or one-way ANOVA with Tukey's post-hoc test (**d**).

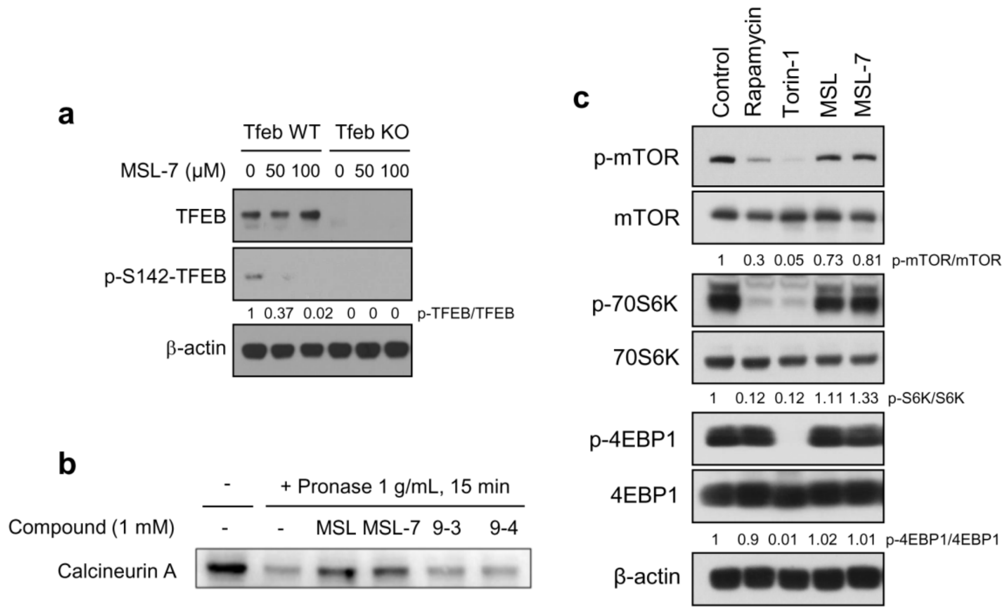

Supplementary Figure 10 TFEB dephosphorylation by MSL-7 and calcineurin A binding. (**a**) *Tfeb* wild-type (WT) and knockout (KO) HeLa cells were treated with MSL-7, and cell extract was subjected to Western blot analysis using the indicated antibodies. (**b**) Cell lysate was treated with pronase with or without pretreatment with MSL, MSL-7 or other MSL derivatives that did not enhance autophagic activity (#9-3 and #9-4), followed by Western blot analysis using anti-calcineurin A antibody. (**c**) HeLa cells were treated with MSL, MSL-7 or controls (rapamycin and Torin-1), and cell extract was subjected to Western blot analysis using the indicated antibodies. Numbers below phospho-S142-TFEB immunoblot bands indicate fold changes normalized to total TFEB bands.

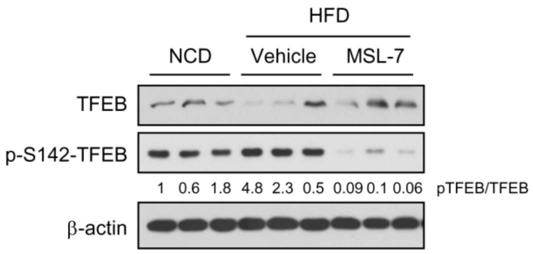

Supplementary Figure 11 Effect of MSL-7 on TFEB phosphorylation *in vivo*. After MSL-7 administration to HFD-fed mice for 8 weeks, liver tissue extract was subjected to Western blot analysis using the indicated antibodies. Numbers below phospho-S142-TFEB immunoblot bands indicate fold changes normalized to total TFEB bands.

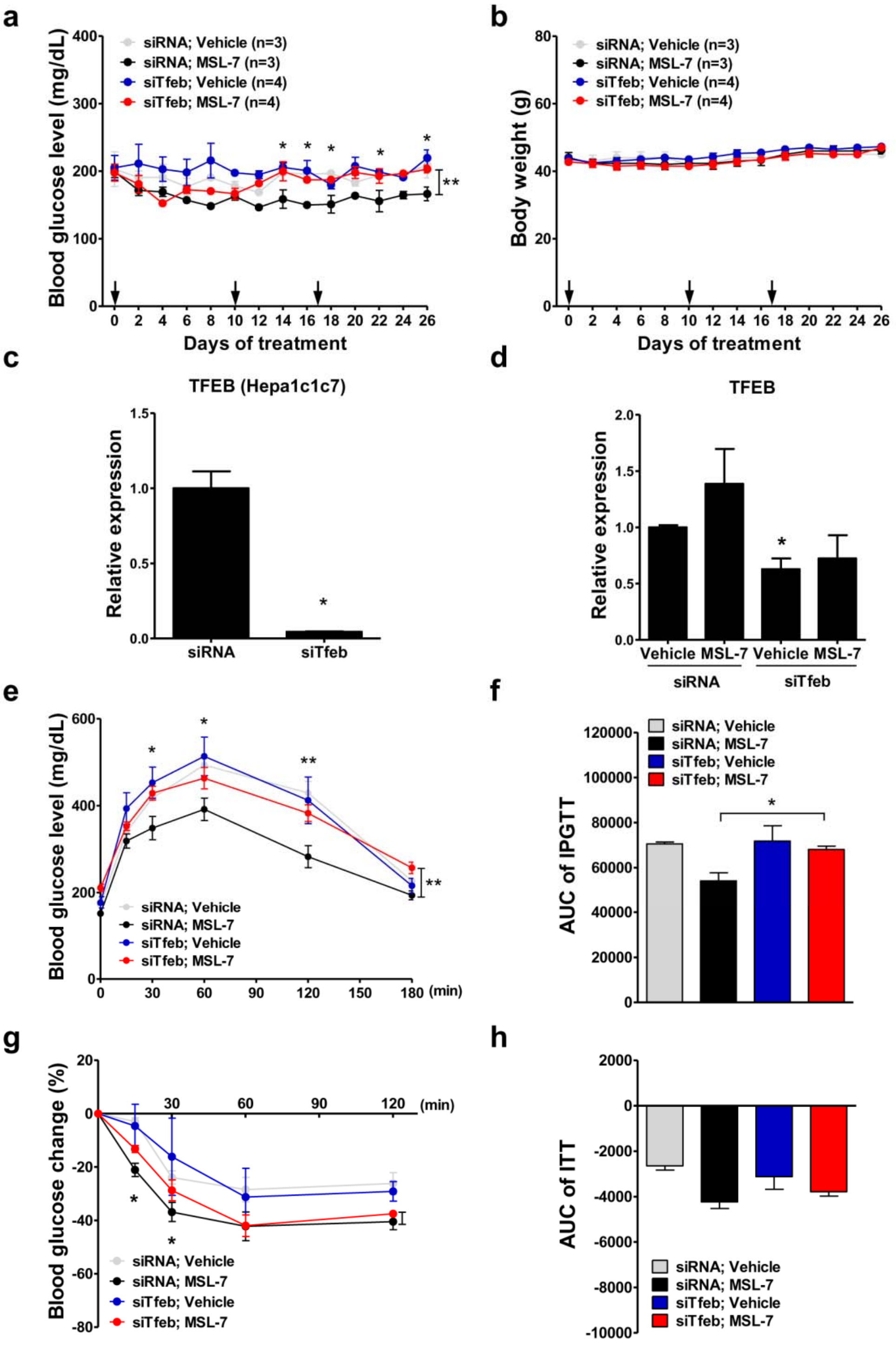

Supplementary Figure 12 Effect of *Tfeb* knockdown on metabolic effects of autophagy enhancer. (**a,b**) Before and during MSL-7 administration to HFD-fed mice, *Tfeb* siRNA (*siTfeb*) was administered to tail vein together with Invivofectamine® 3.0 at the indicated

days (arrows). Nonfasting blood glucose level (**a**) and body weight (**b**) were monitored [F = 18.95, df = 1 for (**a**)]. (**c**) Hepa1c1c cells were transfected with scrambled siRNA (*siRNA*) or si*Tfeb*, and mRNA was isolated for real-time RT-PCR analysis of *Tfeb* expression (t = 8.438, df = 4). (**d**) At the end of experiment, mRNA was isolated from the liver of treated mice, and real-time RT-PCR analysis of *Tfeb* was conducted. (t = 3.26, df = 5). (**e**) IPGTT was conducted after *in vivo* administration of MSL-7 for 8 weeks with or without *in vivo* transfection of *siTfeb* (F = 4.95, F = 10.66, df = 1). (**f**) AUC of the IPGTT curve in (**e**) (F = 4.18, df treatment = 3, df residual = 12). (**g**) ITT was conducted after *in vivo* administration of MSL-7 for 8 weeks with or without *in vivo* transfection of *siTfeb* (F = 5.57, df = 1). (**h**) AUC of the ITT curve in (**g**) (F = 1.83, df treatment = 3, df residual = 12). All data in this figure are the means ± s.e.m. from ≥ 3 independent experiments performed in triplicate. *P < 0.05 and **P < 0.01 by two-way ANOVA with Bonferroni's post-hoc test (**a**), one-way ANOVA with Tukey's post-hoc test (**e**,**f**,**g**,**h**) or two-tailed Student's t-test (**c**,**d**).

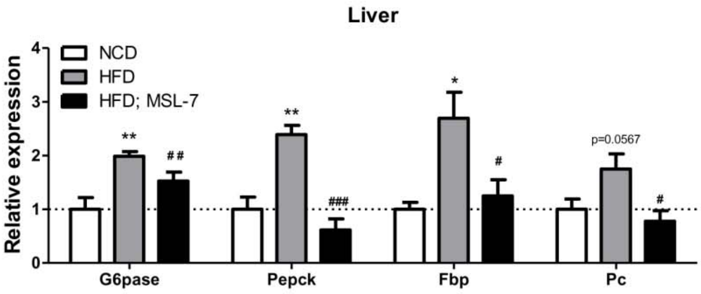

Supplementary Figure 13 The expression of gluconeogenesis genes in HFD-fed mice with or without MSL-7 treatment for 8 weeks. **P* < 0.05 and ***P* < 0.01 by two-tailed Student's *t*-test.

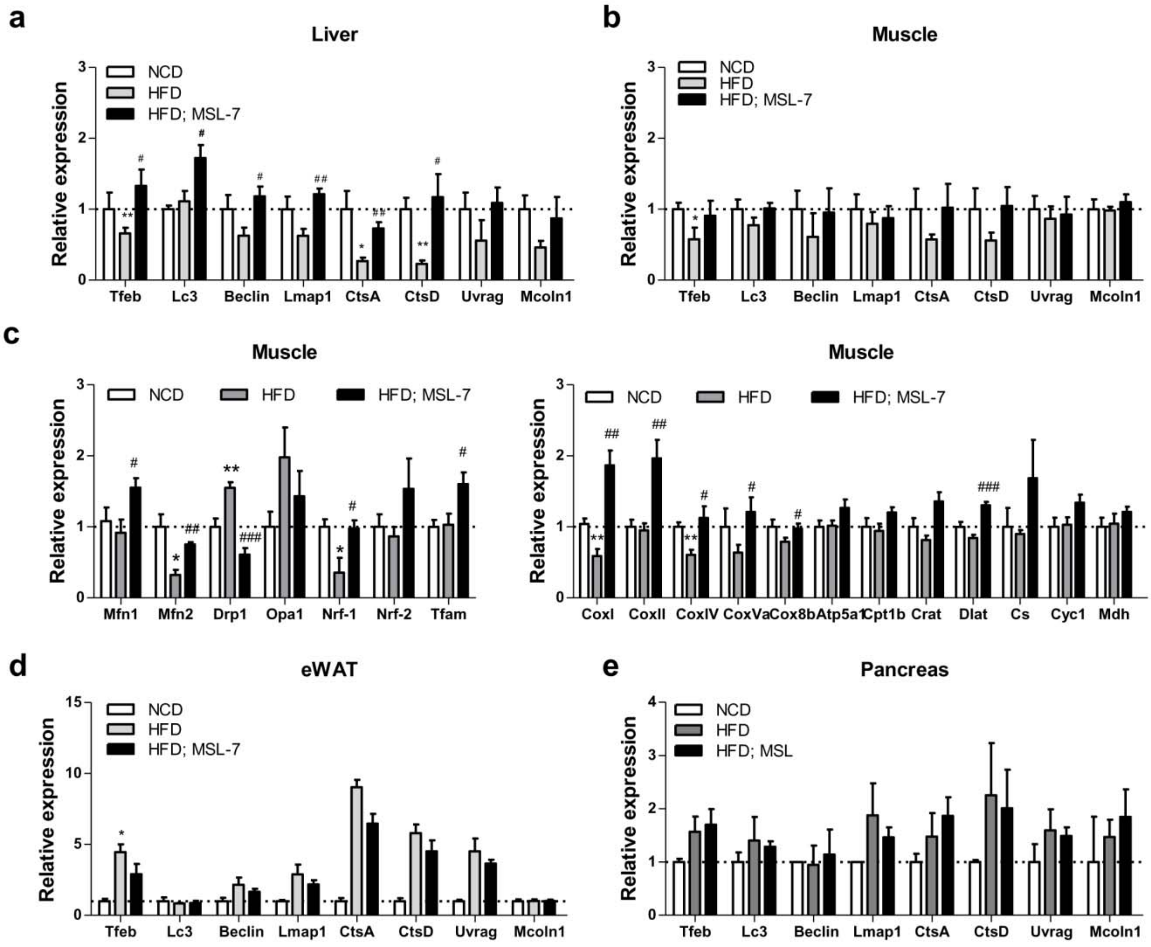

Supplementary Figure 14 The expression of *Tfeb* target genes after MSL-7 administration *in vivo*. mRNA was isolated from HFD-fed mice treated with MSL-7 or vehicle for 8 weeks. The expression of *Tfeb* and its potential target genes was examined by real-time RT-PCR using mRNA from the liver (**a**), skeletal muscle (**b**,**c**), epididymal white adipose tissue (eWAT) (**d**) or pancreas (**e**), and specific primer sets. *P < 0.05 and **P < 0.01 by two-tailed Student's t-test (**a**-**e**).

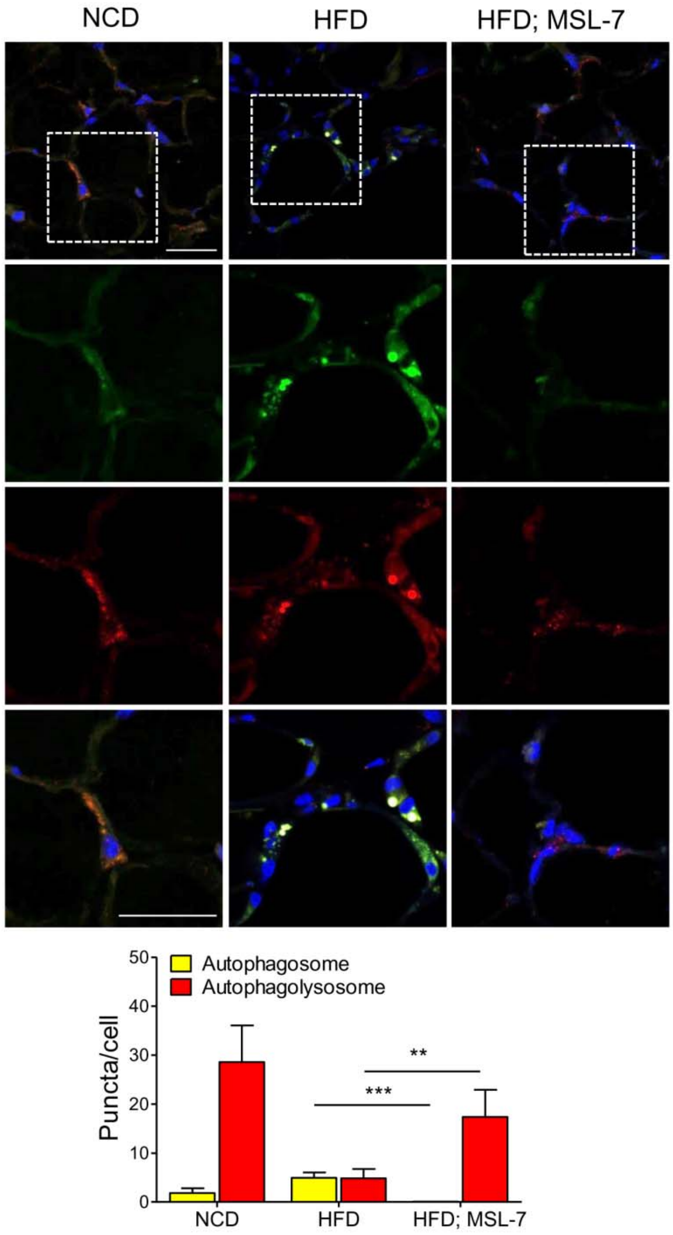

Supplementary Figure 15 Autophagolysosome puncta in epididymal white adipose tissue (eWAT) of mice treated with MSL-7. *CAG-RFP-EGFP-LC3*-transgenic mice were fed HFD for 8 weeks and then treated with MSL-7 or vehicle for additional 8 weeks. The numbers of yellow puncta and red puncta representing autophagosome and autophagolysosome, respectively, were counted. $^{**}P < 0.01$ and $^{***}P < 0.001$ by two-tailed Student's *t*-test.

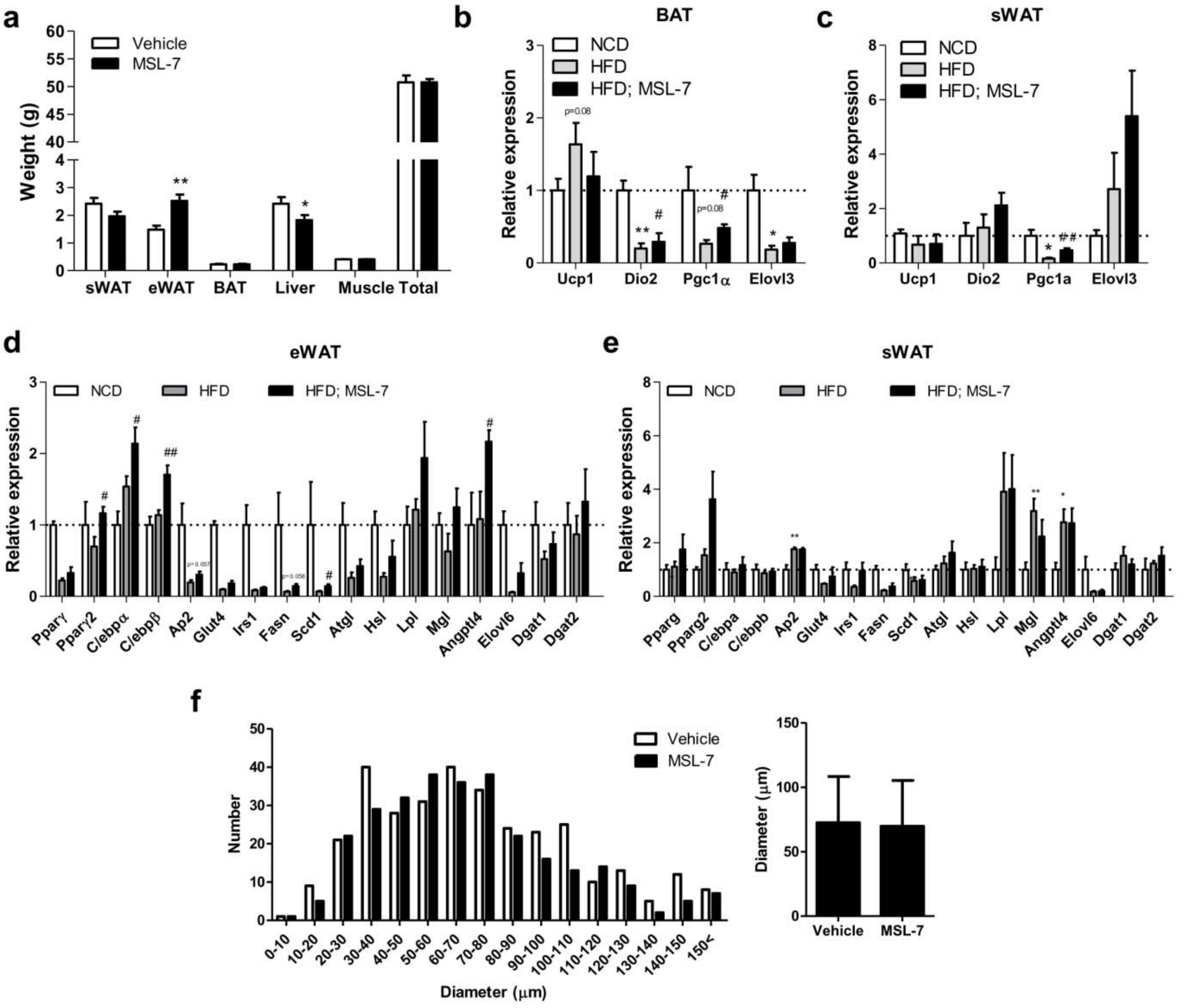

Supplementary Figure 16 The effect of autophagy enhancer on the weight of metabolic organs. (**a**) Weight of each organ was determined after *in vivo* administration of MSL-7 for 8 weeks. (**b-e**) The expression of thermogenesis and lipogenesis genes in adipose tissue. The expression of thermogenesis genes was examined in brown adipose tissue (BAT) (**b**) and subcutaneous white adipose tissue (sWAT) (**c**) of HFD-fed mice treated with MSL-7 for 8 weeks, employing real-time RT-PCR analysis. The expression of lipogenesis genes was examined in epididymal white adipose tissue (eWAT) (**d**) and sWAT (**e**), employing real-time RT-PCR analysis. **(f)** Mean diameter of adipocytes was determined using ImageJ. $*P < 0.05$ and $**P < 0.01$ by two-tailed Student's *t*-test (**a**-**e**).

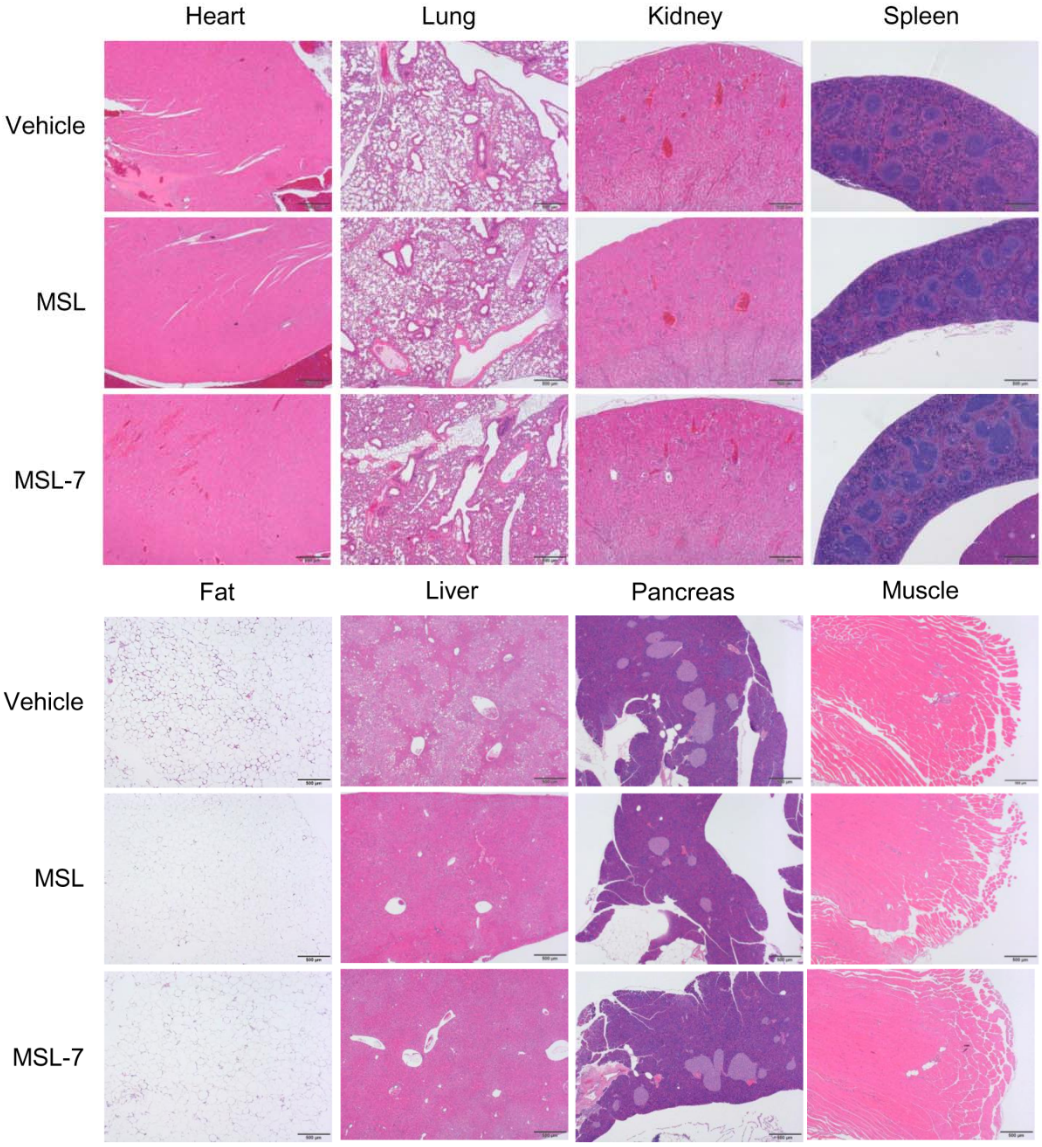

Supplementary Figure 17 No abnormality in the biopsy specimen of major organs after *in vivo* administration of autophagy enhancers. Major organs were obtained from *ob/ob* mice treated with MSL or MSL-7 for 8 weeks, and subjected to histological analysis after H&E staining. No significant change was noted after treatment with MSL or MSL-7 except improvement of fatty liver. (scale bar, 500 μm)

Figure 1.e

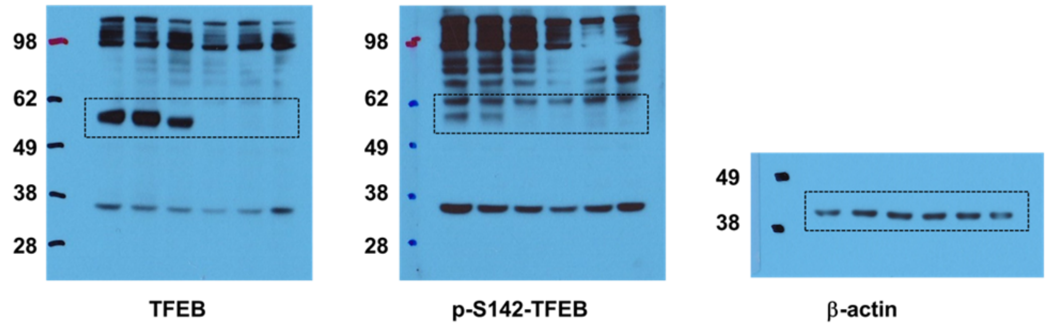

Figure 1.g

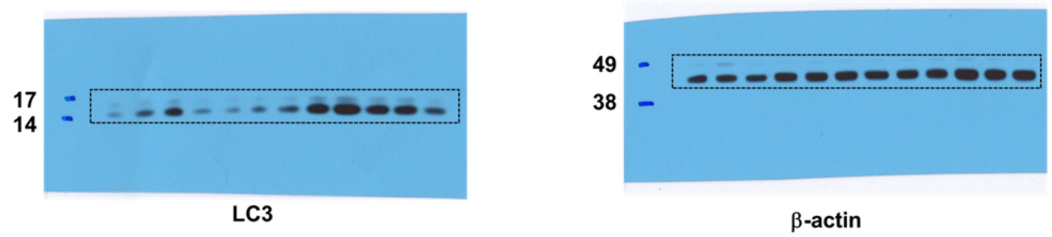

Figure 2.e

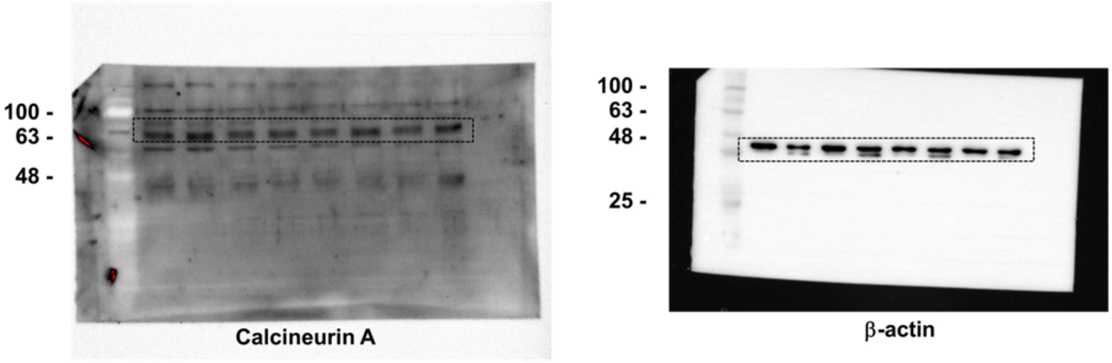

Figure 4.k

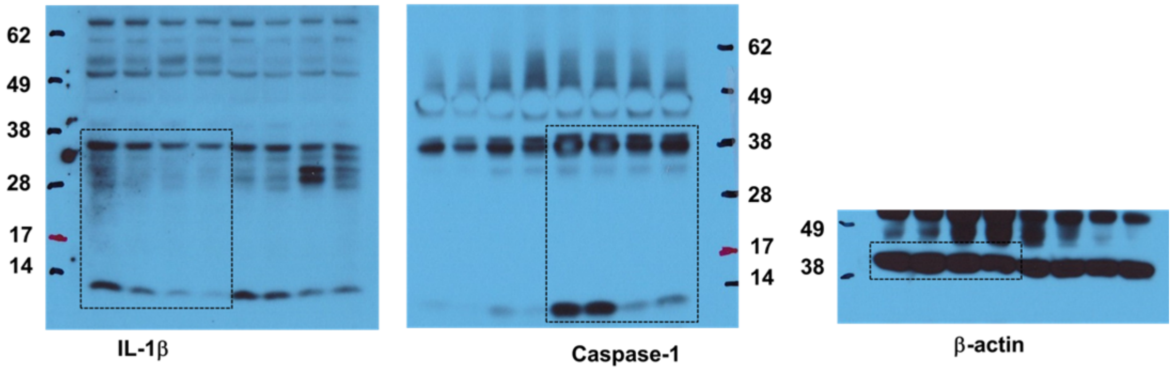

Supplementary Figure 1. d

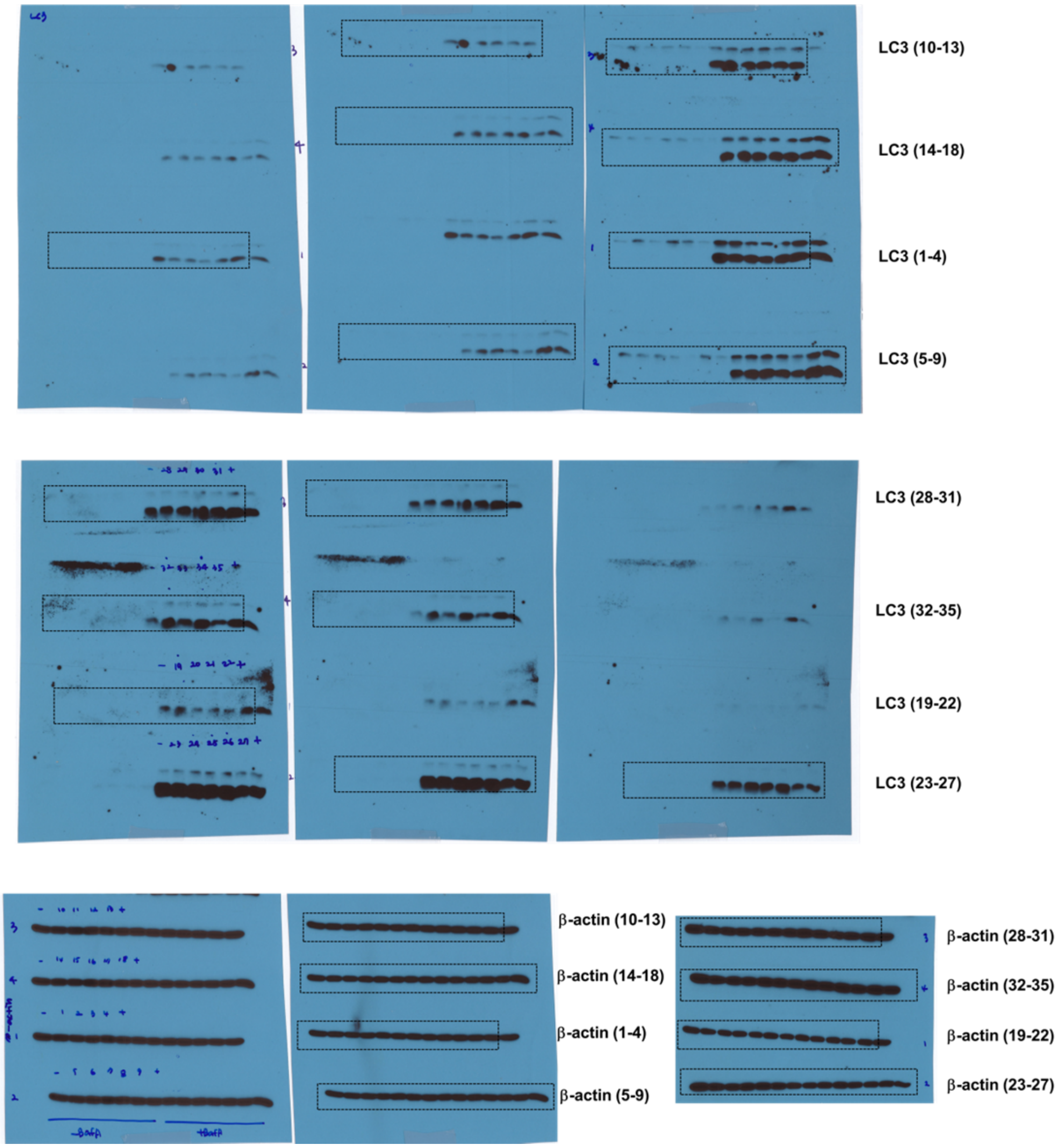

Supplementary Figure 1. e

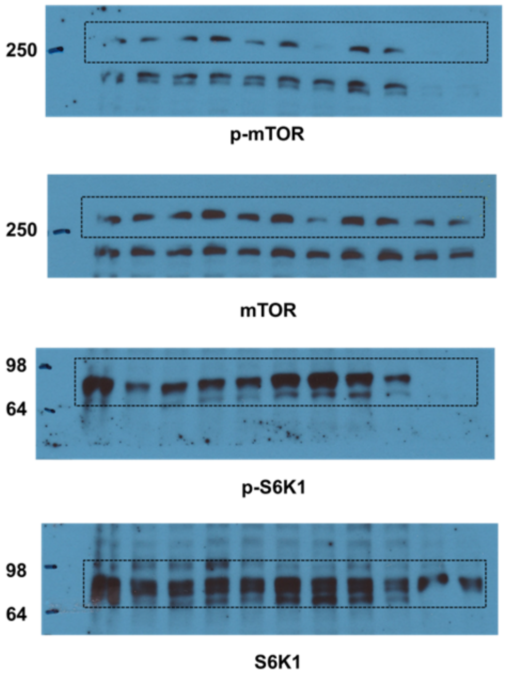

Supplementary Figure 1.c

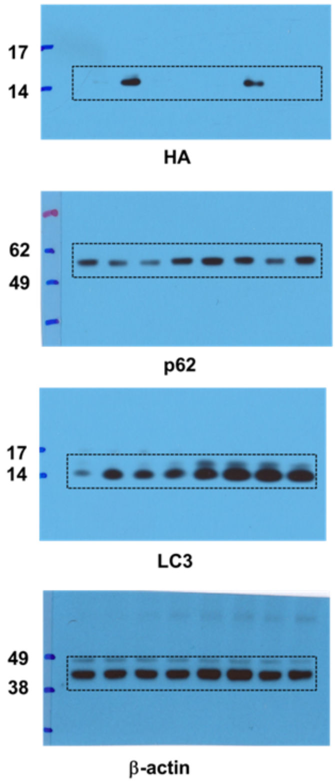

Supplementary Figure 4

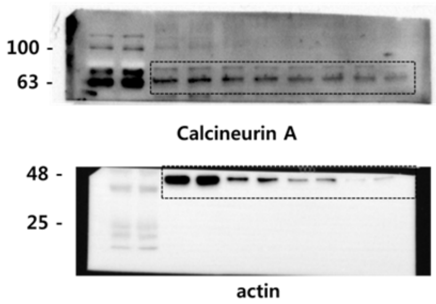

Supplementary Figure 6. b

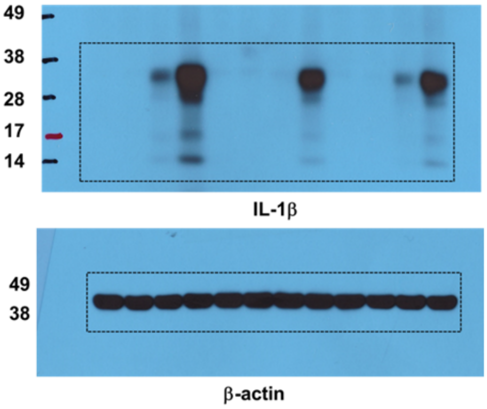

Supplementary Figure 7. b

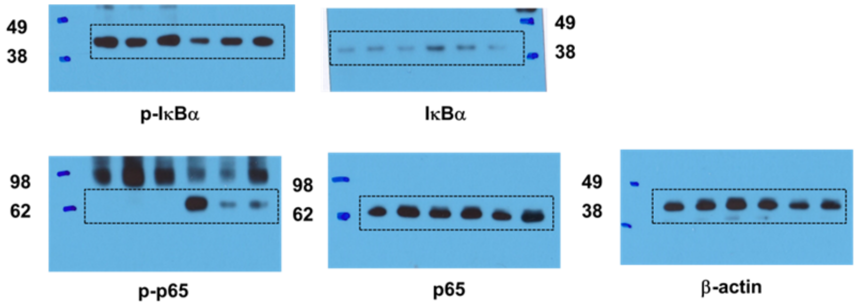

Supplementary Figure 8

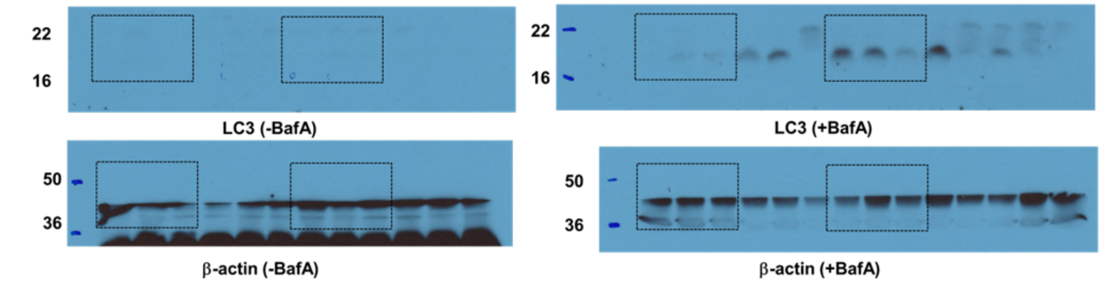

Supplementary Figure 10. a

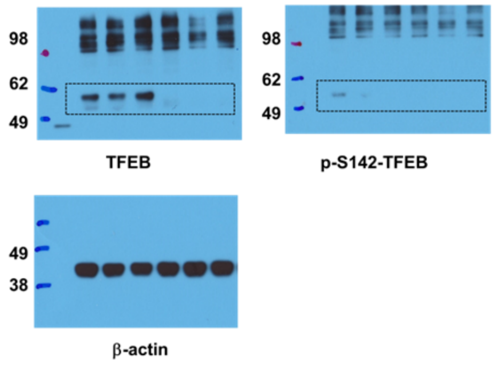

Supplementary Figure 10. b

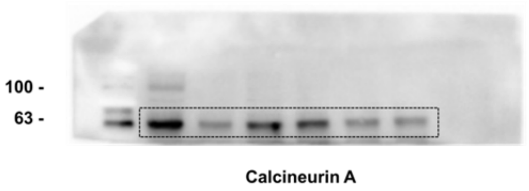

Supplementary Figure 10. c

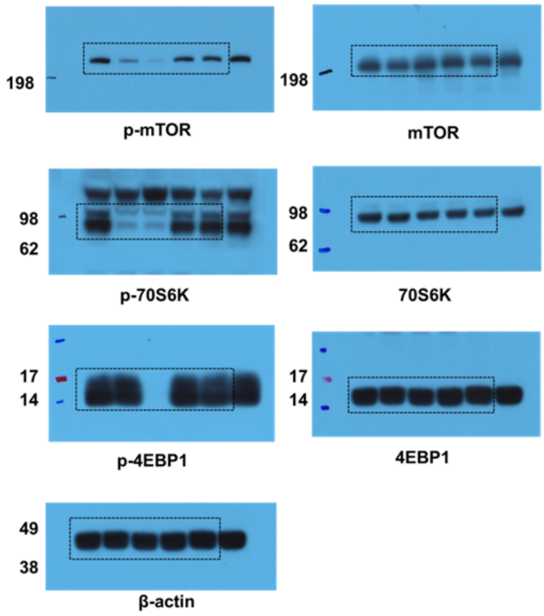

Supplementary Figure 11

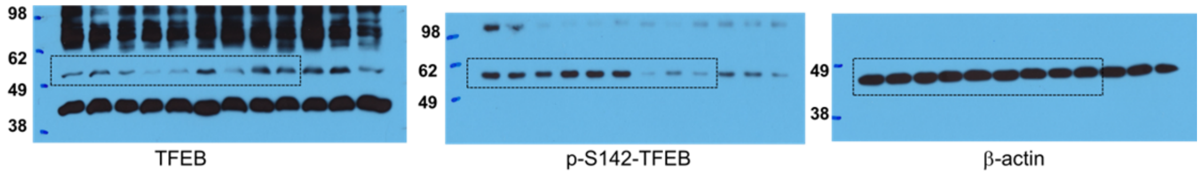

Supplementary Figure 18 Images of original Western blotting.

Supplementary Table 1 Hemogram and blood chemistry after *in vivo* administration of autophagy enhancers for 8 weeks.

Normal Range			Vehicle	MSL	MSL-7
Leukocytes	WBC	1.8-10.7 (K/µl)	6.42 ± 0.78	7.64 ± 1.54	6.26 ± 0.52
	NEU	0.1-2.4 (K/µl)	1.91 ± 0.24	2.34 ± 0.51	1.97 ± 0.16
	LYM	0.9-9.3 (K/µl)	3.93 ± 0.47	4.73 ± 0.95	3.97 ± 0.38
	MONO	0.0-0.4 (K/µl)	0.35 ± 0.08	0.35 ± 0.10	0.28 ± 0.05
	EOS	0.0-0.2 (K/µl)	0.17 ± 0.08	0.165 ±0.06	0.04 ±0.01
	BASO	0.0-0.2 (K/µl)	0.05 ± 0.02	0.05 ± 0.01	0.01 ± 0.01
Erythrocytes	RBC	6.36-9.42 (M/µl)	9.39 ± 0.53	8.82 ± 0.54	9.22 ± 0.12
	HGB	11.0-15.1 (g/dl)	13.86 ± 0.52	14.30 ± 0.56	13.68 ± 0.19
	HCT	38.5-59.0 (%)	50.5 ± 2.4	46.9 ± 2.4	49.25 ± 0.78
Thrombocytes	PLT	592-2972 (K/µl)	926 ± 38	1081 ± 51	1031 ± 55

	Vehicle	MSL	MSL-7
AST (U/l)	222.2 ± 15.30[a]	158.1 ± 16.39[b]	153.8 ± 12.81[b]
ALT (U/l)	281.7 ± 42.52[a]	183.7 ± 35.71[b]	146.9 ± 21.59[b]
TG (mg/dl)	134.4 ± 60.66[a]	113.9 ± 16.41[a]	78.8 ± 22.50[b]
TCHO (mg/dl)	177.2 ± 14.32	150 ± 12.22	136.9 ± 9.73
ALP (U/l)	518.3 ± 57.83[a]	267 ± 35.02[b]	278 ± 26.11[b]
ALB (g/dl)	1.08 ± 0.83	1.1 ± 0.10	1.1 ± 0.10
DBIL (mg/dl)	< 0.5	< 0.5	< 0.5
GGT (U/l)	< 5	< 5	< 5
LDH (U/l)	1848.3 ± 91.79[a]	1429 ± 148.90[b]	810 ± 112.40[c]
CRE (mg/dl)	1 ± 0	0.75 ± 0.14	1 ± 0
CPK (U/l)	336.7 ± 48.7	323.8 ± 29.68	241.3 ± 18.19
CA (mg/dl)	11.17 ± 0.44	11.13 ± 0.24	10.75 ± 0.32
BUN (U/l)	19.17 ± 0.67	21.13 ± 1.38	20 ± 0.89

No adverse changes were noted in hemogram (upper) or blood chemistry (lower) except improved metabolic profile or reduced serum levels of liver enzymes and TG. In this Table, the two groups were labelled with the same alphabet next to the values when the Tukey's post-hoc test after ANOVA did not show a significant difference ($P > 0.05$). Thus, serum TG and LDH level were significantly lower in mice treated with MSL-7 compared to those treated with MSL. (WBC, while blood cell; NEU, neutrophil; LYM, lymphocyte; MONO, monocyte; EOS, eosinophil; BASO, basophil; RBC, red blood cell; HGB, hemoglobin; HCT, hematocrit; PLT, platelet; TG, triglyceride; TCHO, total cholesterol; ALP, alkaline phosphatase; ALB, albumin; DBIL, direct bilirubin; GGT, γ-glutamyltransferase; LDH, lactate dehydrogenase; CRE, creatinine; CPK, creatine phosphokinase; CA, Ca^{2+}; BUN, blood urea nitrogen)

Supplementary Table 2 List of primer sequences used for real-time RT-PCR.

Name	Forwad	Reverse
Tnfα	CCTGTAGCCCACGTCGTAGC	TTGACCTCAGCGCTGAGTTG
Il-6	TTGCCTTCTTGGGACTGATGC	GTATCTCTCTGAAGGACTCTGG
Il-1β	GAATGACCTGTTCTTTGAAGT	TTTGTTGTTCATCTCGGAGCC
F4/80	CTTTGGCTATGGGCTTCCAGTC	GCAAGGAGGACAGAGTTTATCG
Tfeb	GTCATTGACAACATTATGCGCC	GCGTGTTAGGCATCTTGCATCT
Map1lc3b	GCTTGCAGCTCAATGCTAAC	CCTGCGAGGCATAAACCATGTA
Beclin	AGGCTGAGGCGGAGAGATT	TCCACACTCTTGAGTTCGTCAT
Lamp1	CCTACGAGACTGCGAATGGT	CCACAAGAACTGCCATTTTTC
Ctsa	TTCTGATCCAGCCAGATGGTG	TACAGCACGTTGGCAATCAGG
Ctsd	CGTCCTTTGACATCCACTACGG	TGGAACCGATACAGTGTCCTGG
Uvrag	CAAGCTGACAGAAAAGGAGCGAG	GGAAGAGTTTGCCTCAAGTCTGG
Mcoln1	GCGCCTATGACACCATCAA	TATCCTGGCACTGCTCGAT
G6pase	GCTGGAGTCTTGTCAGGCAT	ATCCAAGCGCGAAACCAAAC
Pepck	GATCATCATGCACGACCCCT	CCGGAACCAGTTGACATGGA
Pc	GGGATGCCCACCAGTCACT	CATAGGGCGCAATCTTTTTGA
Fbp1	ACTTGATCCCCAGTCACATTG	CGATCAAAGCCATCTCGTCT
Mfn1	TGTGTTCGGATTTTCAAGAGGACA	CTCCTGGGCTGCATTATCCG
Mfn2	CATGTCCACGATGCCCAAC	GACAAAGTGCTTGAGAGGGG
Drp1	AGGAGATGCAGAGGATCATTCAG	ATCAGCAAAGTCGGGGTGTT
Opa1	ATGATTGGGCCAGACTGGAA	AGGTAAGCTGGGTGCTCATC
Nrf-1	CAGACACGTTTGCTTCGGAAA	CCCACTCGCGTCGTGTACT
Nrf-2	GATCAGGCGACATGTTAACGTT	AGAGCCCAGTCAAACCCTTTC
Tfam	AGATATGGGTGTGGCCCTTG	AAAGCCTGGCAGCTTCTTTG
CoxI	TGCTAGCCGCAGGCATTACT	CGGGATCAAAGAAAGTTGTGTTT
CoxII	CAGGCCGACTAAATCAAGCAA	GAGCATTGGCCATAGAATAATCCT
CoxIV	TCACTGCGCTCGTTCTGAT	CGATCGAAAGTATGAGGGATG
CoxVa	TCATCCAGGAACTTAGACCAACT	AGTCCTTAGGAAGCCCATCG
Cox8b	CGAAGTTCACAGTGGTTCCC	GCTGGAACCATGAAGCCAAC
Atp5a1	GACAGTACCCTCCTTCCACC	ACCAGCAAGAACAGGGTAAGA
Cpt1b	GACAGTACCCTCCTTCCACC	ACCAGCAAGAACAGGGTAAGA
Crat	GGAGAAGAGAGCCAGTCCAG	AGATAATCCTCCCACCGCTG
Dlat	TGTTTCATCGGTGTTGAGTCTG	TGCTAATGATGTGCCCTTGTG
Cs	GACCCTCGCTATTCCTGTCA	AGTTCATCTCCGTCATGCCA
Cyc1	GGCTCCTCCCATCTACACAG	CTGACCACTTATGCCGCTTC
Mdh	TGAACGGGAAGGAAGGAGTC	AATGCCCAGGTTCTTCTCCA
Usp1	TATCATCACCTTCCGCTG	GTCATATGTTACCAGACTCTG
Dio2	GGGACTCCTCTCTGTCTTTT	CCAACTTCGGACTTCTTGTA
Pgc1a	TCTGGAACTGCAGGCCTAACTC	GCAAGAGGGCTTCAGCTTTG
Elovl3	CGTAGTCAGATTCTGGTCCT	CCAGAAGAAGTGTTCCGTTG
Pparγ	ATCCCTGGTTTCATTAACCT	GCTCCATAAAGTCACCAAAG
Pparγ2	TGGGTGAAACTCTGGGAGAT	CCATAGTGGAAGCCTGATGC
C/ebpα	GCGCAAGAGCCGAGATAAAG	CGGTCATTGTCACTGGTCAACT
C/ebpβ	CGCCTTTAGACCCATGGAAG	CCCGTAGGCCAGGCAGT
Ap2	ACATGAAAGAAGTGGGAGTG	GAAGTCACGCCTTTCATAAC
Glut4	ACAATGTCTTGGCTGTGCTG	TCCCACATACATAGGCACCA
Irs1	TGGATGCAAGTGGATGACTC	CGGAGGATTGTTGAGATGGT
Fasn	CTCCGTGGACCTTATCACTA	CTGGGAGAGGTTGTAGTCAG
Scd1	TGGAAATGCCTTTGAGATGG	CCAGCCAGCCTCTTGACTAT
Atgl	CATGATGGTGCCCTATACTC	GTGAGAGGTTGTTTCGTACC
Hsl	AAGGACTTGAGCAACTCAGA	TTGACTATGGGTGACGTGTA
Lpl	ATGGAGAGCAAAGCCCTGCTC	ATACTCAAAGTTAGGCCCAGC
Mgl	GACGGACAGTACCTCTTTTG	AGAAAAGTAGGTTGGCCTCT
Angptl4	GTTTGCAGACTCAGCTCAAGG	CCAAGAGGTCTATCTGGCTCTG
Elovl6	CCCGAACTAGGTGACACGAT	TACTCAGCCTTCGTGGCTTT
Dgat1	ACAGGGAAGAAGGTCAGTGG	AGGGGAACGCTCACTAGGTA
Dgat2	TCATGGGTGTCTGTGGGTTA	CAGAGTGAAACCAGCCAACA
S18	AGGTTCTGGCCAACGGTCTAG	CCCTCTATGGGCTCGAATTTT
